# A Versatile Method for Preparing Polysaccharide Conjugates via Thiol-Michael Addition

**DOI:** 10.3390/polym13121905

**Published:** 2021-06-08

**Authors:** Junyi Chen, Xutao Ma, Kevin J. Edgar

**Affiliations:** 1 College of Polymer Science and Engineering, Qingdao University of Science and Technology, Qingdao 266042, China; 15552266138@163.com; 2 Department of Sustainable Biomaterials, Virginia Tech, Blacksburg, VA 24061, USA; kjedgar@vt.edu

**Keywords:** polysaccharide chemical modification, thiol-Michael addition, cationic polysaccharide

## Abstract

Polysaccharide conjugates are important renewable materials. If properly designed, they may for example be able to carry drugs, be proactive (e.g., with amino acid substituents) and can carry a charge. These aspects can be particularly useful for biomedical applications. Herein, we report a simple approach to preparing polysaccharide conjugates. Thiol-Michael additions can be mild, modular, and efficient, making them useful tools for post-modification and the tailoring of polysaccharide architecture. In this study, hydroxypropyl cellulose (HPC) and dextran (Dex) were modified by methacrylation. The resulting polysaccharide, bearing α,β-unsaturated esters with tunable DS (methacrylate), was reacted with various thiols, including 2-thioethylamine, cysteine, and thiol functional quaternary ammonium salt through thiol-Michael addition, affording functionalized conjugates. This click-like synthetic approach provided several advantages including a fast reaction rate, high conversion, and the use of water as a solvent. Among these polysaccharide conjugates, the ones bearing quaternary ammonium salts exhibited competitive antimicrobial performance, as supported by a minimum inhibitory concentration (MIC) study and tracked by SEM characterization. Overall, this methodology provides a versatile route to polysaccharide conjugates with diverse functionalities, enabling applications such as antimicrobial activity, gene or drug delivery, and biomimicry.

## 1. Introduction

Polysaccharide conjugates have proved to be valuable in drug delivery [1], gene delivery [2], as a vaccine carrier [3], and in many other biomedical applications. Functionalization of polysaccharides is always challenging; approach angles are restricted, diffusion is slow, and discrimination between similarly unreactive groups is a major challenge. Hence, the development of new methods for functionalizing polysaccharides is an important task. In recent decades, “click chemistry” methods have been developed that share attractive features (e.g., rapid kinetics, mild conditions, high efficiency, high yield) and some have been applied to polysaccharides [4]. Azide-alkyne Huisgen cycloaddition [5], olefin cross-metathesis [6,7,8], and the thiol-ene reaction [9,10], are among those reported to have been applied to polysaccharides [6,11,12], affording a broad range of pathways for designing bioconjugates.

Thiol-Michael addition is mild, versatile and efficient, and is even more suitable for polysaccharide modification since it is tolerant to water and air. It is frequently used in the preparation of polysaccharide hydrogels, due to its typically rapid kinetics in water. Feijen [13] reported the modification of dextran with a vinyl sulfone, and used it as an acceptor for thiol-Michael addition to prepare degradable hydrogels. Albertsson [14] investigated the synthesis of thiolated hemicellulose and its use in hydrogel preparation. Cabrera [15] reported the thiolation of chitosan, and synthesized several chitosan-antimicrobial peptide conjugates. Although a number of polysaccharide hydrogels were designed and prepared by thiol-Michael addition, there are only a few studies of limited scope that discuss its usage in the synthesis of polysaccharide conjugates [16]. Due to the typically benign nature of polysaccharides, and the in vivo biodegradability of properly chosen polysaccharides, their conjugates have advantages over conventional synthetic polymers for biopharmaceutical uses [4]. Thiol-Michael reactions of polysaccharides typically fall short of “click” characteristics only in that an excess of the small molecule reactant is frequently useful to drive the reaction; this excess small molecule reagent should be readily removed from the product polymer. We expect this approach to be a great chemistry tool for tailoring the architecture of polysaccharide, and thus deserving of further investigation. Thus, thiol-Michael chemistry is anticipated to be very useful for functionalizing polysaccharides, and quite worthy of detailed exploration.

Polymers bearing positive charges exhibit many useful physical and chemical properties. Chitosan, which is derived from chitin, is perhaps the most important cationic polysaccharide with antimicrobial activity [17,18]. At pH values below 6–6.5, the protonated glucosamine units of chitosan provide cationic character, and enable its interaction with bacterial membranes, followed by the acetyl motif permeabilization. Despite its promising biocompatibility with some tissues and in some situations, its DS(Ac)-dependent biodegradability, and the aforementioned antimicrobial properties, the positive charge and solubility of chitosan rely strongly on pH, seriously restricting its applications. Quaternary ammonium groups are permanently positively charged, and therefore may exhibit pH-insensitive antimicrobial performance, assisting the absorption of bacteria. As a result, the hydrophobic linker to the ammonium cation, usually an alkyl, can readily destroy the cell membrane and eliminate the bacterium. Therefore, introducing quaternary ammonium salts to polysaccharides has attracted the attention of investigators. Domb [2] reported the preparation of quaternary ammonium-substituted dextran by reductive amination. This approach requires periodate oxidation of dextran vicinal diols, thus compromising its stability and degrading the dextran’s physical properties. Stanciu [19] reported the synthesis of cationic dextran derivatives by a similar approach, and the resulting material exhibited good antimicrobial activity toward *S. aureus* and *Candida parapsilosis*. In a separate approach, quaternary amines were appended by radical graft copolymerization from hydroxyethyl cellulose, creating a quaternized cellulose composite hydrogel with antimicrobial and hemostatic properties [20]. Chitin [21] and chitosan [22] were also investigated as quaternized polysaccharides for similar purposes. In addition, cationic polysaccharides can interact with negatively charged macromolecules to form polyelectrolyte complexes, which are of interest for applications like gene delivery [2] and as mRNA carriers in vaccines [3].

Zwitterionic polysaccharides (ZPCs) possess both anionic and cationic groups with overall charge neutrality, with the potential for applications including in contact lenses [23], medical devices [24], biosensors [25], and implantable medical materials [24]. Moreover, zwitterionic polymers have shown high resistance to non-protein adsorption. Due to this fact, zwitterionic polymers can avoid quick recognition by the immune system and exhibit delayed blood clearance from the body, promising features for long-duration drug delivery [26,27]. ZPCs have also been reported being used as immunomodulatory agents capable of activating a T-cell-dependent immune response in the absence of protein [28]. Dextrans have been frequently used as low-cell and protein-binding substrates, including in column chromatography applications and cell culture microcarrier bead technology [29]. Few routes to zwitterionic polysaccharides have been reported, thus we were motivated to consider pathways to zwitterionic dextran derivatives [30].

Our goal was to develop and demonstrate an efficient synthetic route for preparing polysaccharide conjugates via thiol-Michael addition, and briefly examine their properties and potential applications. We chose two polysaccharide-based starting materials: dextran and hydroxypropyl cellulose. Dextran is an important natural polysaccharide which is frequently used in biomedical applications. It is biocompatible in many situations, and the amylase enzyme can recognize and degrade the dextran *in vivo*. Dextran with a molecular weight less than 30,000 g/mol can pass into the urine through the kidney microtubules. Hydroxypropyl cellulose is a commercial cellulose ether that is commonly used for pharmaceutical formulations in which clearance from the circulation is not required, including as a tablet binder and in artificial tear lubricants. It is an economical and low-toxicity material which is thermally processable. Both of these polysaccharides are good substrates for designing functional polysaccharide conjugates, and they each have solubility not only in water, but in certain organic solvents, permitting easier chemical modification. We hypothesized that both dextran and HPC could be readily methacrylated in organic solution [31], and that those methacrylates would be excellent acceptors for the conjugate addition of thiols, via click-like thiol-Michael addition. We further hypothesized that these efficient thiol-Michael additions would be effective methods for imparting useful new functionality to polysaccharides. Herein we test these hypotheses by attempting the proposed methacrylation reactions, then exploring the thiol-Michael reactivity of the products toward several reaction partners, including 2-thioethyl amine, cysteamine, and a quaternary ammonium thiol. We investigate the antimicrobial activity of polysaccharide conjugates bearing quaternary ammonium salts, and seek to understand their interesting and useful properties.

## 2. Experimental Section

### 2.1. Materials

Bis(2-dimethylaminoethyl) disulfide dihydrochloride was purchased from TCI (Tokyo, Japan), and dextran and hydroxypropyl cellulose (DS(HP) = 2.2, MS(HP) = 4.4, M_w_ = 10,000 g/mol) were purchased from Shanghai Dibo Biotechnology Co. (Shanghai, China) Methacrylate anhydride, triethyl amine, cysteine, ethylamine hydrochloride, DTT, and DMSO were purchased from Shanghai Energy Chemicals Co. (Shanghai, China) Dialysis tubing (MWCO 3500 g/mol) was purchased from Beijing Wokai Biotechnology Co. (Beijing, China).

### 2.2. Measurements

The ^1^H NMR spectra were acquired on a Bruker (Billerica, MA, USA) AV500 FT-NMR spectrometer. All samples were analyzed as solutions in D_2_O or d6-DMSO at 25 °C in standard 5 mm o.d. tubes. The size distribution and zeta potential of dextran conjugate aqueous solution (1 mg/mL) were measured by a Malern Zetasizer (Westborough, MA, USA) Nano ZS90 instrument with a quartz cuvette. Polysaccharide samples were dissolved in distilled water at a concentration of 1 mg/mL. The measurement was carried out at 25 °C and repeated for 3 trials. The FT-IR spectra of polysaccharide samples were collected on a Bruker tensor 27 FT-IR spectrometer. The samples were ground into powders with KBr, and were then pressed into pellets.

### 2.3. Preparation of Dex-MA and HPC-MA

Dextran (1.5 g) was dissolved in 20 mL deionized water or DMSO, then triethylamine (0.056 g, 0.55 mmol) and methacrylic anhydride (0.84 g, 5.45 mmol, 0.2 equiv/agu) were added slowly. The solution was stirred at 60 °C (water) or 25 °C (DMSO) overnight. Subsequently, the solution was dialyzed against deionized water for 48 h. Finally, the purified Dex-MA was freeze-dried to give 1.5 g product (DS(MA) = 0.1, determined by ^1^H NMR, yield 96%). HPC-MA was prepared in the exact same manner, with a 97% yield.

Dex-MA: ^1^H NMR (D_2_O): δ 1.97 s, [-C(CH_3_)=CH_2_], δ 3.0–4.2 m, (H2–H6 of dextran backbone), δ 4.99 d, (anomeric proton), δ 5.79 s, 6.24 s, [-C(CH_3_)=CH_2_].

HPC-MA: ^1^H NMR (D_2_O): δ 1.14 d, [-CH_2_-CH(-CH_3_)-O-], 1.92 s, [-C(CH_3_)=CH_2_], 2.13 s, (CH_3_-CH(CH_3_)-OH), 3.0–4.0 m, (H2-H6 of HPC backbone), [-CH_2_-CH(-CH_3_)-O-], [-CH_2_-CH(-CH_3_)-O-], 4.26 bs, ((-O-CH_2_-CH(CH_3_)OH), anomeric proton), 5.73 s, 6.14 s, [-C(CH_3_)=CH_2_].

### 2.4. Preparation of N-ethyl-N,N-dimethy-2-thioethyl Aminium Iodide (QA-SH)

Bis(2-dimethylaminoethyl) disulfide dihydrochloride (3.0 g, 10.6 mmol) was dissolved in aqueous 1 N NaOH (3.0 mL). The free base, bis[2-(N,N-dimethylamino)ethyl] disulfide was then extracted into dichloromethane (100 mL), dried, concentrated in a vacuum and used directly without further purification. To a stirred solution of the resulting bis[2-(N,N-dimethylamino)ethyl] disulfide in 20 mL acetonitrile, ethyl iodide (8 mL, 99.5 mmol) was added dropwise over a period of 15 min, and the reaction contents were further stirred for one hour at room temperature. The precipitates formed were filtered, washed with cold acetonitrile, then diethyl ether, and then dried to give a white solid (yield 88.7%):

^1^H NMR (D_2_O): δ 1.38 t, [-CH_2_-CH_3_], 3.11 s, [-CH_3_], 3.15 t, [-S-CH_2_-CH_3_], 3.45 q, [-CH_2_-CH_3_], 3.68 t, (-S-CH_2_-CH_2_-).

The resulting product was dissolved in 10 mL methanol, followed by adding DL-Dithiothreitol (DTT) (0.5 g, 3.2 mmol). The solution was stirred overnight, then was precipitated in excess diethyl ether, filtered, and dried in a vacuum to offer a sticky liquid-like product (yield: 83.6%):

^1^H NMR (D_2_O): δ 1.36 t, [-CH_2_-CH_3_], 2.92 q, [-CH_2_-CH_2_-SH], 3.07 s, [-CH_3_], 3.41 q, [-CH_2_-CH_3_], 3.49 t, (HS-CH_2_-CH_2_-).

### 2.5. Procedure for Thiol-Michael Addition of Dex-MA and HPC-MA with 2-Thioethyl Amine (EA), Cysteine (Cys), and N-ethyl-N,N-dimethy-2-thioethyl Aminium Iodide (QA-SH)

Dex-MA (0.4 g, DS(MA) = 1.22, determined by ^1^H NMR) was dissolved in 15 mL DMSO under nitrogen. QA-SH (0.48 g, 2.0 equiv.) and triethyl amine (0.45 mL, 2.0 equiv.) were added. After 1 h, the solution displayed a brown color. The reaction solution was then added to dialysis tubing (MWCO, 3500 g/mol), then dialyzed against deionized water for 48 h. The final product was obtained by freeze-drying, giving a light yellow cotton-like solid (yield 94%). Dex-g-Cys (yield 95%) and Dex-g-EA (91%) were prepared by equivalent procedures. The thiol-Michael addition of HPC-MA was carried out under the same conditions and molar ratio. Note that the reaction can take place in water if the substrate is water soluble.

Dex-QA: ^1^H NMR (D_2_O): δ 1.28 d, [-CH(CH_3_)-], 1.37 t, [-CH_2_-CH_3_], 3.02 t, [-S-CH_2_-CH_2_-], 3.07 s, [-CH_3_], 3.3–4.2 m, (H2–H6 of dextran backbone), [-CH_2_-CH_3_], (HS-CH_2_-CH_2_-), 4.99 d, (anomeric proton).

HPC-QA: ^1^H NMR (D_2_O): δ 1.17 d, [-CH_2_-CH(-CH_3_)-O-], 1.37 t, [-CH_2_-CH_3_], 2.19 d, [-CH(CH_3_)-O-], 3.3–4.2 m, (H2–H6 of dextran backbone), [-CH_2_-CH_3_], (HS-CH_2_-CH_2_-), 4.43 bs, ((-O-CH_2_-CH(CH_3_)OH), anomeric proton).

Dex-EA: ^1^H NMR (D_2_O): δ 1.15 d, [-CH(CH_3_)-], 2.74–3.15 m, [-CH(CH_3_)-], [-S-CH_2_-CH-], [-CH_2_-CH_2_-S-], [NH_2_-CH_2_-CH_2_-], 3.3–4.2 m, (H2–H6 of dextran backbone), 4.99 d, (anomeric proton).

Dex-Cys: ^1^H NMR (D_2_O): δ 1.15 d, [-CH(CH_3_)-], 2.72 m, [-CH(CH_3_)-], 3.12 [-S-CH_2_-CH-], [-CH-CH_2_-S-], 3.3–4.2 m, (H2–H6 of dextran backbone), [-CH(NH_2_)-COOH], 4.99 d, (anomeric proton).

### 2.6. Minimum Inhibitory Concentration

The dextran conjugates were tested for the minimum inhibitory concentration against *E. coli* and *S. aureus*. Polysaccharide samples in Mueller Hinton broth were incubated with equal volumes of bacterial suspension (10^6^ CFU/mL). MIC showed the lowest concentration in the sample, where complete inhibition of bacteria growth was determined by a microplate reader.

### 2.7. SEM Characterization

SEM characterization was used to demonstrate the antimicrobial performance of polysaccharides conjugated with quaternary ammonium salt (*E. coli* pH 7.4 PBS with CFU 10^6^). Then the polysaccharide samples were dissolved in the solution with a concentration at 10 mg/mL, then stirred at 37 °C for 8 h. The SEM samples were prepared by placing drops of the solution on a silicon film, then the solution was dried at 37 °C overnight. The samples were sputtered with platinum before characterization. The surface profile of *E. coli* was obtained from Quanta FEG 250 SEM operated at an accelerating voltage of 10 kV.

## 3. Results and Discussion

### 3.1. Synthesis of Polysaccharide Substrates and Their Thiol-Michael Addition Products

**Scheme 1:**
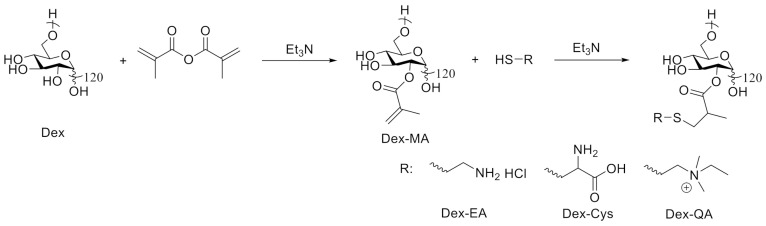
Synthesis of Dex-MA, and its thiol-Michael addition products. (Methacrylation is not regioselective).

**Scheme 2:**
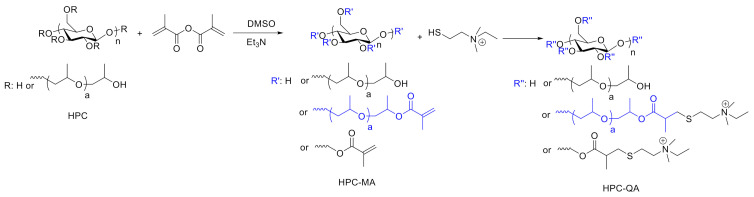
Synthesis of HPC-MA and HPC-QA.

In order to prepare polysaccharide conjugates, we synthesized Dex-MA (Scheme 1) and HPC-MA (Scheme 2) with a range of DS(MA) (Table 1). Dex-MA is an ideal substrate for three reasons:

1. Dextran is so non-toxic that it is routinely used in intravenous infusions in the clinic, and is biodegraded in vivo and cleared through the kidneys once it reaches the appropriate DP, thus dextran derivatives have in vivo application potential;
2. Dex-MA is soluble in several solvents, including DMSO, DMF, and water, providing a broad leeway for further chemical modification. The fact that Dex-MA with low DS(MA) (<0.4) is water soluble also makes it suitable for modification under green conditions;
3. Targeted degrees of MA substitution can be achieved quantitatively, allowing for tailoring to different application requirements.

**Figure 1:**
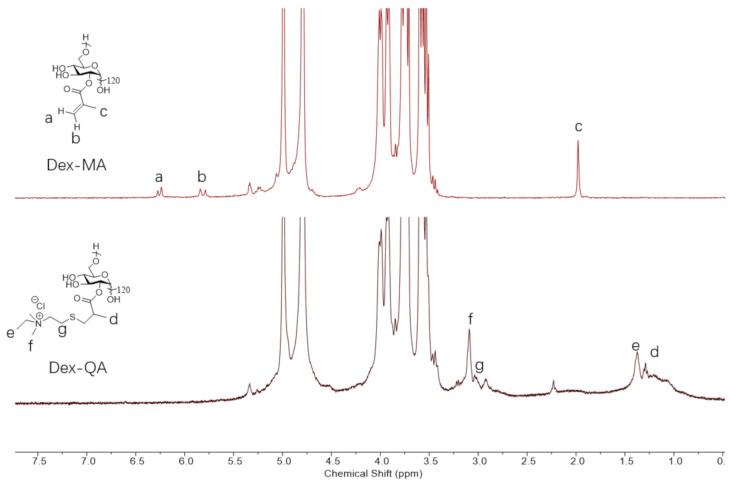
^1^H-NMR spectra of DEX-MA and DEX-QA.

**Figure 2:**
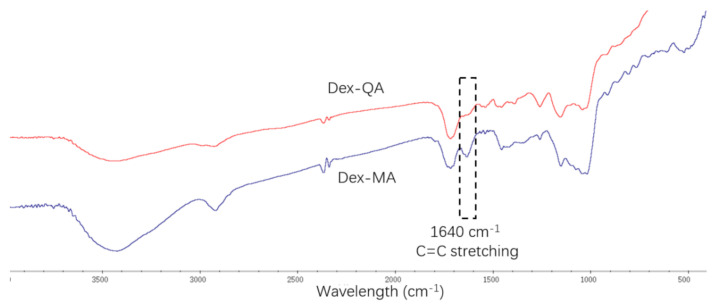
FT-IR spectra of Dex-QA and Dex-MA.

We used a simple esterification method to introduce methacrylate (MA) to dextran, in which triethylamine was used as the base catalyst and methyl acrylate anhydride as the esterification reagent [32], in either DMSO or water. Chemical structures and DS values of the Dex-MA and HPC-MA samples could be determined by ^1^H NMR (Figure S3). As shown in Figure 1, resonances from the methacrylate motif appeared clearly at 6.24 ppm (a) and 5.78 ppm (b), and the peak at 1.97 ppm was assigned to the methyl (c), well separated from the dextran backbone; all were consistent with the proposed structure. To demonstrate that Dex-MA has a tunable DS(MA), dextran was reacted with different equivalents of methacrylate anhydride under different conditions. The DS(MA) was calculated based on the integrals of isolated olefin resonances with that of the dextran anomeric H (1). Table 1 displays that Dex-MA with a different DS(MA) can be prepared. We discovered that Dex-MA with high DS(MA) could be only obtained at a high reaction temperature (70 °C) with DMSO as solvent. Using water as solvent afforded water-soluble Dex-MA with relatively small DS(MA). Note that a DS(MA) below 0.4 was necessary to preserve the water-solubility of Dex-MA. The proposed Dex-MA chemical structure is also supported by FT-IR. As shown in Figure 2, a distinctive absorbance at 1640 cm^−1^ was observed and assigned as an acrylate C=O stretch.

In this study, hydroxypropyl cellulose (HPC), a commercially available cellulose ether frequently used in biomedical applications, was selected as another example. The methacrylation were very similar to those used with dextran, and HPC-MA was also synthesized as another thiol-Michael addition substrate (Scheme 2).

With a series of Dex-MA and HPC-MA at hands, we then explored the synthesis of their conjugates. Thiol-Michael addition is a simple and versatile reaction that can be carried out with suitable polysaccharide acceptors under mild, click-like conditions (room temperature, short reaction time, generally near-quantitative conversion [33]), in the presence of an organic base catalyst. It falls short of click criteria in that an excess of the small molecule reagent is frequently needed to afford the desired rapid kinetics [34]. We realized that this highly efficient method might provide a straightforward pathway to polysaccharide conjugates. We expect these substrates would undergo clean thiol-Michael addition with thiol-functionalized molecules.

In order to test the hypothesis, further explore and demonstrate the application of Dex-MA conjugates, we synthesized a cationic dextran derivative, Dex-QA. QA-SH was reacted with Dex-MA, using a thiol-Michael addition process directly analogous to those used to make Dex-EA and Dex-Cys. The introduction of quaternary ammonium salts to the dextran would potentially endow the polysaccharide derivative with antimicrobial properties. Unlike other antimicrobial polysaccharides like chitosan, the permanent cations of Dex-QA should be capable of interacting with bacterial membranes at a broad range of pH values, and the hydrophobic alkyl groups (methyl and ethyl) groups may help destroy the bacterial membrane and eliminate the bacterium. Several studies have reported [2,22] the modification of polysaccharides with quaternary ammonium salt. This, however, appears to be the first report of the introduction of quaternary ammonium salts to a polysaccharide via thiol-Michael addition. Moreover, this is a general method that fits for most polysaccharides without destroying backbone structure. The thiol-quaternary ammonium salt, QA-SH (Scheme S1, Figures S4 and S5), was synthesized based on a reported method [35]. Thiol-Michael addition was carried out in either DMSO or H_2_O, with triethyl amine as catalyst, and the resulting product was purified by dialysis against deionized water. The chemical structure of Dex-QA was determined by ^1^H-NMR spectroscopy (Figure 1). The appearance of resonance at 1.37 ppm (e), and 3.08 (f) ppm, and the disappearance of the olefin resonances, suggest that conjugate addition occurred with an essentially quantitative conversion, affording the desired thiol-grafted and cationic product. Dex-QA was also characterized by FT-IR (Figure 2). The distinctive C=C stretching absorbances at 1640 cm^−1^ from starting Dex-MA olefin disappeared after thiol-Michael addition, supporting the formation of Dex-QA.

**Figure 3:**
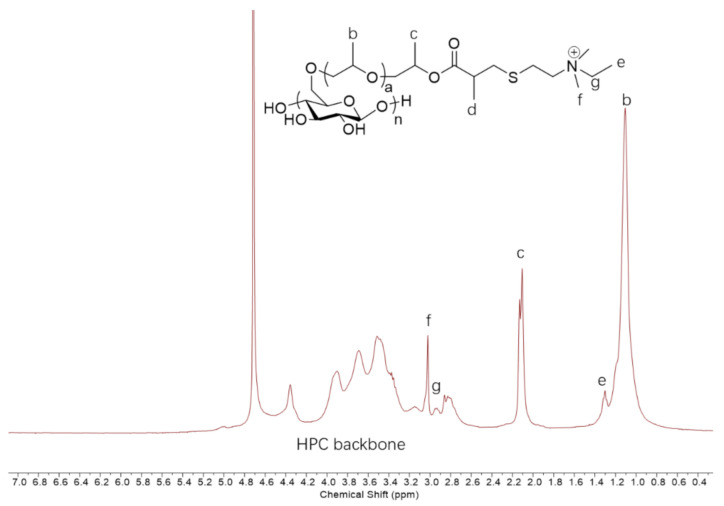
^1^H-NMR spectrum of HPC-QA.

HPC-MA was also a promising thiol-Michael addition substrate, particularly since the methacrylates were mostly at the ends of oligo (HP) chains, as we predicted, and therefore would have wider approach angles. HPC-MA was reacted with QA-SH by a procedure (Scheme 2) closely analogous to that used for methacrylated dextran. The chemical structure of the resulting HPC-QA was determined by ^1^H NMR. As shown in Figure 3, the disappearance of the conjugated olefin resonances, and the appearance of peaks at 1.36, 3.01, and 3.09 ppm, contributed by protons from the quaternary ammonium salt, are clearly observed, suggesting that the proposed HPC-QA structure was achieved.

After the successful thiol-Michael addition of Dex-MA and HPC-MA with QA-SH, we knew that the polysaccharide-bearing methacrylate is a type of efficient thiol-Michael addition substrate, which motivated us to continue to explore its scope and potential utilities. We hypothesized that molecules with a thiol motif would be effective reagents in this grafting strategy. This synthetic route likely affords access for designing various polysaccharide conjugates, including those with biomedical application potentials.

To further explore the substrate, the thiol-Michael addition of Dex-MA was carried out in water at room temperature, using 2-thioethyl amine as a nucleophile. In this polymer–small molecule graft reaction, it was simple to purify the product by dialysis against deionized water, as all unconsumed catalyst and excess reagents would be washed away. The ^1^H NMR spectrum of Dex-g-EA (Figure S1) illustrated that the reaction was complete within 1 h, with 100% conversion, determined as described below. In the starting Dex-MA (Figure 1), resonances from olefin protons appeared clearly at 6.24 ppm (a) and 5.78 ppm (b). Upon thiol-Michael addition, the olefin was consumed and the peaks disappeared, indicating complete conversion to the targeted product. The appearance of resonances at 3.07 ppm and 2.92 ppm, assigned to the ethylene of the ethyl amine, supported the proposed product structure. Dex-MA with high DS(MA) (short for degree of substitution of methacrylate) also approached 100% conversion.

We proceeded to extend the scope of this class of material; we selected cysteine, a semi-essential proteinogenic amino acid, as a reaction partner. The thiol side chain in cysteine can react with Dex-MA, using a thiol-Michael addition process directly analogous to that used to make Dex-EA and Dex-QA. The incorporation of cysteine to dextran can examine the feasibility of preparing dextran–amino acid, and even dextran–protein, conjugates. It may also open up an approach for glycoprotein mimicking. Furthermore, as cysteine has a pair of amine and carboxylic acid, the Dex-Cys is a zwitterionic polymer, which may have interesting properties, including antifouling and pH responsiveness. The synthesis of Dex-Cys is very similar to that of Dex-EA. The product chemical structure and the thiol-Michael addition conversion was determined by ^1^H NMR (Figure S2), which clearly showed the disappearance of isolated olefin resonance, and the appearance of a peak at 2.89 ppm, which is contributed by the methylene from the cysteine. This outcome suggests that dextran–amino acid conjugates can be achieved by this approach. We expect this strategy can be applied to the preparation of polysaccharide-peptide conjugates. Thus, applications like drug delivery, tissue engineering and immunomodulatory agents can be developed based on this functional polysaccharide.

### 3.2. Zeta Potential of Polysaccharide Conjugates

It was of great interest to study the zeta potential of these polysaccharide conjugates. The zeta potential values provide information about the charge characteristics of the polysaccharide conjugates. As the polysaccharide conjugates are all water soluble, their aqueous solutions can be analyzed by dynamic light scattering (DLS). We started with the HPC conjugates since HPC itself displays neutral zeta potential (0.4 mV) in distilled water. Just as expected, appending QA onto the HPC polymer chain increased its zeta potential (+14 mV), confirming that HPC-QA is a cationic polymer, which is promising for its antimicrobial performance. The charge characteristics of HPC-Cys, a zwitterionic polymer, is investigated under different pH environments (pH = 3, 7, 11). The HPC-Cys displays a positive zeta potential (+3.8 mV) under acidic conditions, and negative zeta potential under neutral (−1.6 mV) or basic (−6.2 mV) conditions, consistent with the expected behavior of a zwitterionic polymer.

The dextran used in this study displays a negative zeta potential (−16 mV) in distilled water, which is probably due to a small degree of oxidation of primary or anomeric alcohols to carboxylates, presumably during polymer isolation or storage. The inherent negative charge offsets the introduction of cations. Therefore, we mainly discuss the charge characteristics of HPC conjugates.

### 3.3. Antimicrobial Activity of Polysaccharide Quaternary Ammonium Salt Conjugates

To evaluate their antimicrobial activity, the minimum inhibitory concentration (MIC) of HPC-QA, Dex-QA, and chitosan were investigated. MIC is the lowest concentration of a compound that inhibits visible growth of a bacteria. Chitosan with 75% deacetylation was used as a positive control, as it is a known antimicrobial polysaccharide. According to the MIC results, the polysaccharide samples have antimicrobial activity against *S. aureus* (Gram-positive) and *E. coli* (Gram-negative). We found that the quaternary ammonium salt conjugates have better inhibitory effects on Gram-positive bacteria than that of Gram-negative strains. This behavior agrees with many other quaternary ammonium polymer studies [36]. Among these samples, HPC-QA exhibited the lowest MIC (1.25 mg/mL) toward *S. aureus*. Dex-QA and chitosan had similar *S. aureus* MIC, which were 3.75 and 5.00 mg/mL, respectively. The *E. coli* MIC values of HPC-QA, Dex-QA, and chitosan against *E. coli* were 3.75, 7.5 and 7.5 mg/mL, respectively. A lower MIC value indicates a better antimicrobial performance. These results support the hypothesis that the introduction of quaternary ammonium salts by thiol-Michael addition can impart considerable antimicrobial activity to polysaccharide conjugates’ antimicrobial properties.

**Figure 4:**
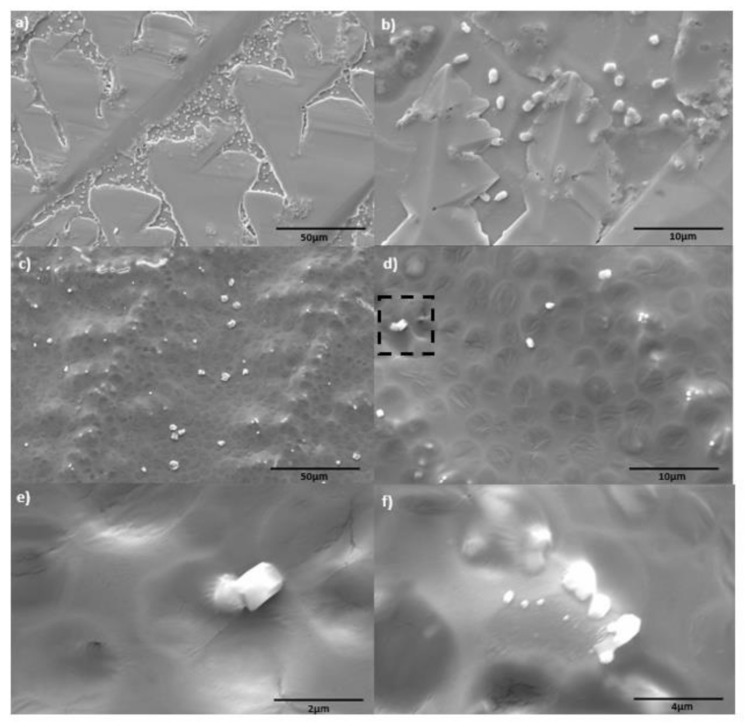
SEM images of HPC-QA/*E. coli* PBS sample (**c**–**f**) and *E. coli* PBS sample (**a**,**b**). (**e**) is the enlarged region labeled in (**d**).

SEM permits the direct observation of bacterial morphology. If bacteria lose their original shape (consistent with cell membrane destruction), it suggests that the bacteria were killed. *E. coli* was selected for this experiment due to its easy availability and clear rod shape, which can be easily observed in SEM images. The HPC-QA (DS(QA) = 1.12) was dissolved in the *E. coli* pH 7.4 PBS and stirred for 8 h, allowing the cationic polysaccharide to fully interact with the bacteria. The resulting solution was casted onto the top of silicon films and sputtered with platinum, to make them ready for SEM characterization. As shown in Figure 4a,b, the control (raw *E. coli* PBS) displays a great number of 2 μm rod-shaped *E.coli.* In contrast, the SEM images of the HPC-QA/*E. coli* sample show limited amounts of bacteria. Moreover, some of the fractured bacteria can be observed (Figure 4e,f), suggesting the quaternary ammonium salt successfully attached to and destroyed the *E. coli* cell membranes. This supports the hypothesis that the incorporation of QA gives the polysaccharide conjugates antimicrobial properties. The SEM images also show an interesting behavior of HPC-QA; the cationic polysaccharide seems to prevent salt in the solution from forming large crystalline domains.

### 3.4. Solubility

**Figure 5:**
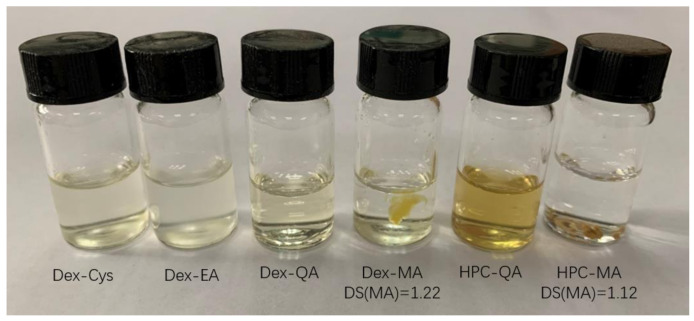
Water solubility of dextran and HPC derivatives (from left to right, Dex-Cys, Dex-EA, Dex-QA, Dex-MA, HPC-QA and HPC-MA).

The water solubility of a given polysaccharide conjugate is very important for its potential in biomedical applications, such as in hydrogels, drug or gene delivery systems, or as antimicrobial agents. As mentioned above, the introduction of methacrylate reduces the hydrophilicity of dextran, and Dex-MA with high DS(MA) displays poor water solubility, especially when DS(MA) exceeds 1.0 (Figure 5). Due to the high water affinity of thiol-Michael addition partners (ethylamine, cysteine, and QASH), the resulting dextran and HPC conjugates, even with high DS(MA), show good water solubility (Figure 5), suggesting that these hydrophilic motifs were chemically appended onto the polysaccharide-MA substrates and had the designed impact on properties. The good water solubility of these polysaccharide conjugates is quite beneficial to their application potentials.

## 4. Conclusions

We have demonstrated the concept of thiol-Michael addition to appended methacrylates as an efficient and versatile approach for preparing polysaccharide conjugates. The chosen polysaccharide substrates, Dex-MA and HPC-MA, have good solubility in water and organic solvents. Moreover, their DS(MA) can be readily tuned, and various thiol-containing molecules can be grafted onto these substrates with essentially quantitative conversion, and under mild (click-like) conditions. This is the first time that a quaternary ammonium salt was grafted onto polysaccharides via thiol-Michael addition, thereby creating promising sustainable antimicrobial materials. We also discovered that this synthetic strategy is a good approach for coupling polysaccharides with thiol-containing amino acids, opening up a pathway for designing polysaccharide conjugates with amino acid and polypeptides.

In this work, we prepared the polysaccharide conjugates Dex-EA, Dex-Cys, Dex-QA, HPC-QA, and HPC-Cys through this grafting strategy. Their chemical structures were confirmed by ^1^H NMR and FT-IR spectroscopic techniques. The charge characteristics of polysaccharide conjugates were investigated by DLS in dilute aqueous solution, which suggested that the polysaccharide derivative zeta potentials can be rationally designed by choosing different thiol-Michael addition reaction partners. The polysaccharides bearing quaternary ammonium salts display good antimicrobial properties against Gram-negative *E. coli* and Gram-positive *S. aureus*, which are supported by the MIC and SEM studies. This synthetic strategy should be applicable to most other polysaccharides as well. It creates access to a wide range of functionalized, polysaccharide-based graft copolymers, in high conversion and under mild conditions. Such new, sustainable materials, created from benign natural polysaccharides, should prove particularly useful for preparing and designing drug/gene delivery systems, hydrogels, and in other biomedical applications.

## Figures and Tables

**Table 1:** Preparation of Dex-MA with different DS(MA).

Equivalents (Methacrylate Anhydride/AGU)	Temperature (°C)	Solvent	DS(MA)
1.0	25	H_2_O	0.00
1.0	70	H_2_O	0.03
0.2	25	DMSO	0.10
0.37	25	DMSO	0.30
0.50	70	DMSO	0.42
2.0	70	DMSO	1.22

## Data Availability

Not applicable.

## Supplementary Materials

The following are available online at https://www.mdpi.com/article/10.3390/polym13121905/s1. Figure S1. ^1^H NMR spectrum of Dex-EA; Figure S2. ^1^H NMR spectrum of Dex-Cys; Scheme S1. Synthesis of N-Ethyl-2-thiol-N,N-dimethylethan-1-aminium iodide (QA-SH); Figure S3. Calculation of the DS(MA) of Dex-MA; Figure S4. ^1^H NMR spectrum of QAS-SQA; Figure S5. ^1^H NMR spectrum of QA-SH.
