# Supplementary material for: A Versatile Method for Preparing Polysaccharide Conjugates via Thiol-Michael Addition
Source: Polymers (Basel). 2021 Jun 8;13(12):1905. doi: 10.3390/polym13121905 (PMC8228737; doi:10.3390/polym13121905)
Supplement: Supplementary file 1 [file polymers-13-01905-s001.zip › polymers-1236790-supplementary.pdf]

polymers

MDPI

Supplementary Materials: A versatile method for preparing polysaccharide conjugates via thiol Michael addition

Junyi Chen[1,*], Xutao Ma[1] and Kevin J. Edgar[2]

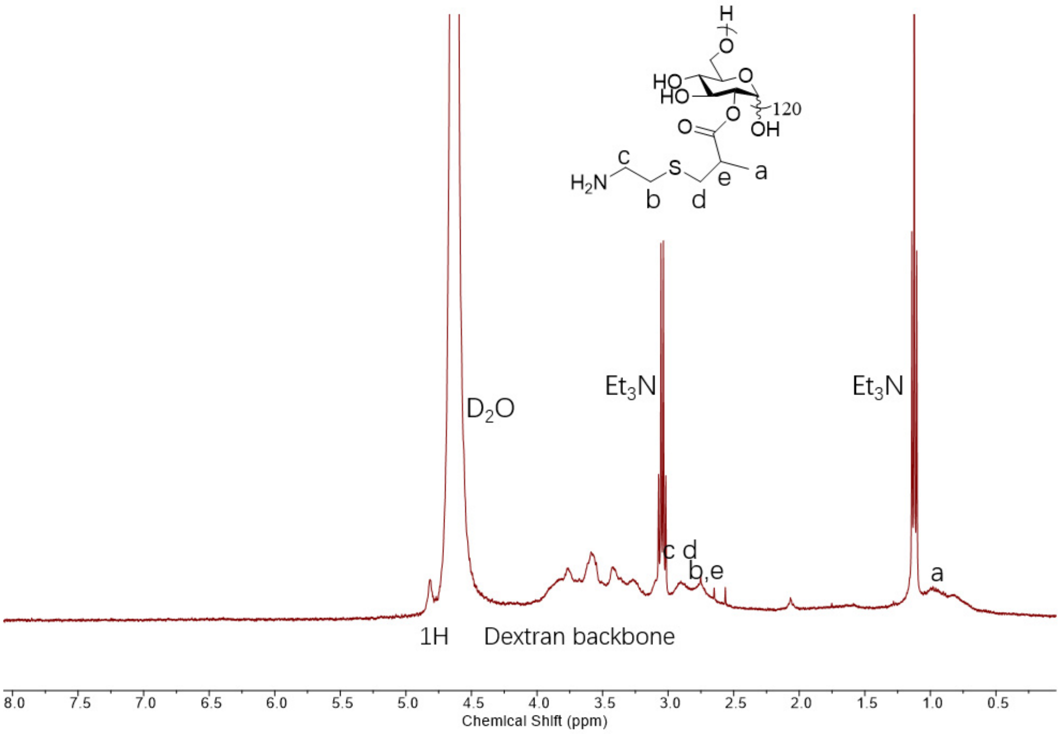

Figure S1. ^1H NMR spectrum of Dex-EA.

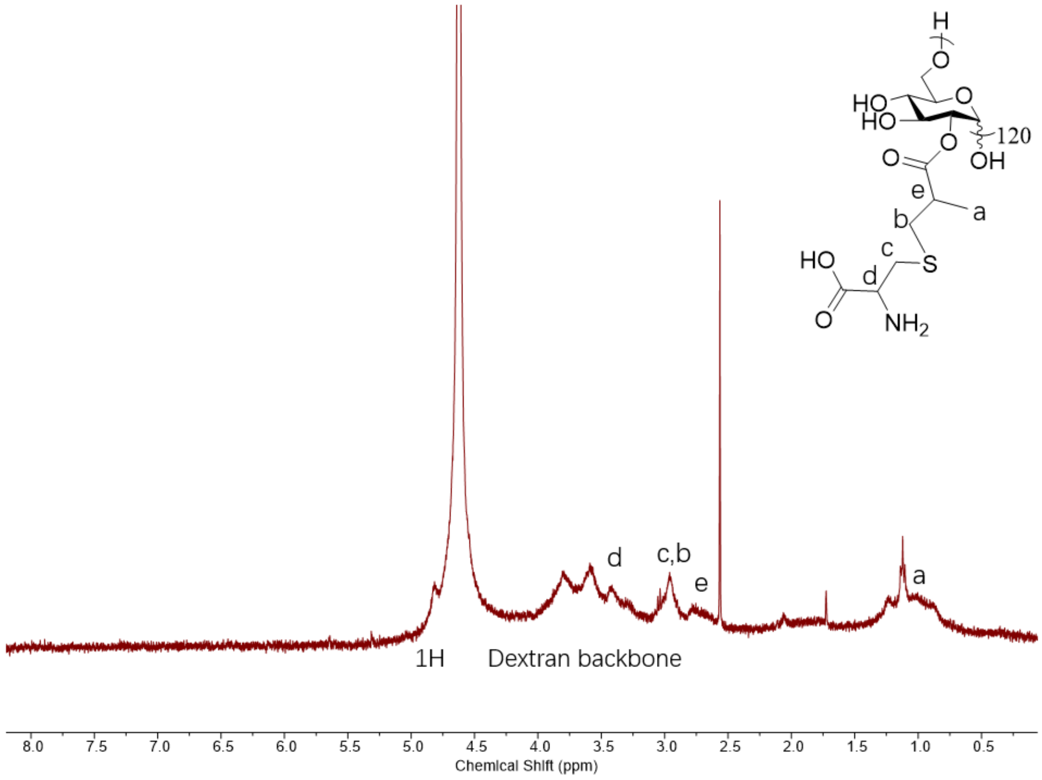

Figure S2. ^{1}H NMR spectrum of Dex-Cys.

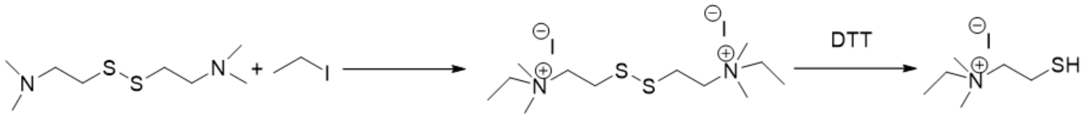

Scheme S1. Synthesis of N-Ethyl-2-thiol-N,N-dimethylethan-1-aminium iodide (QA-SH).

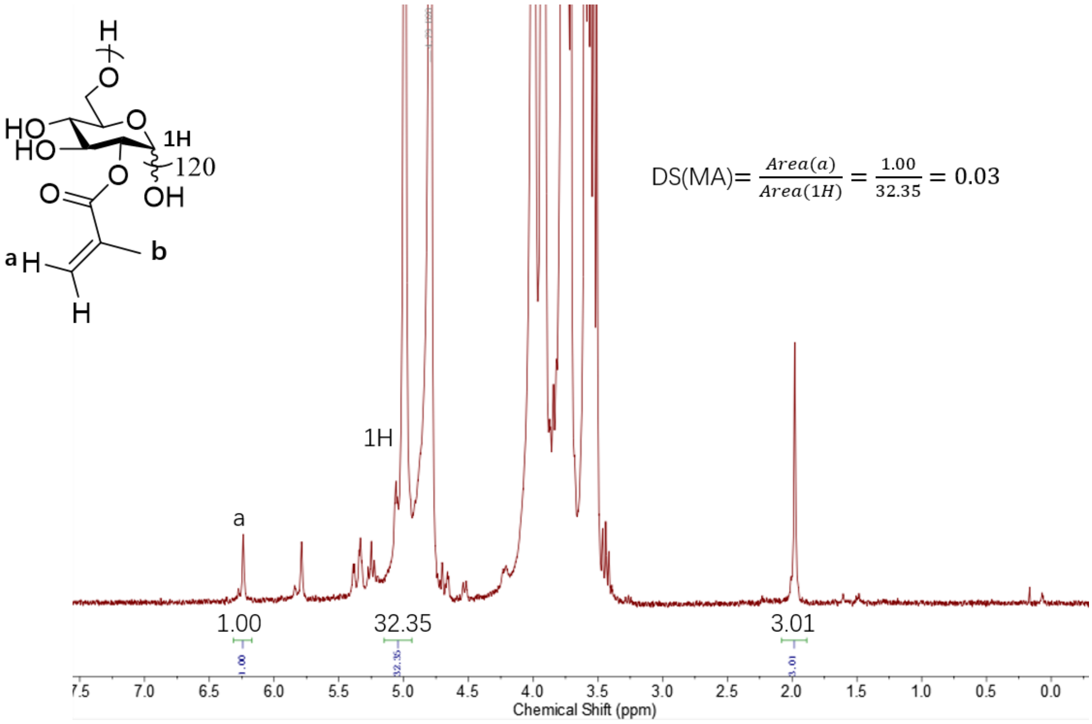

Figure S3. Calculation of the DS(MA) of Dex-MA.

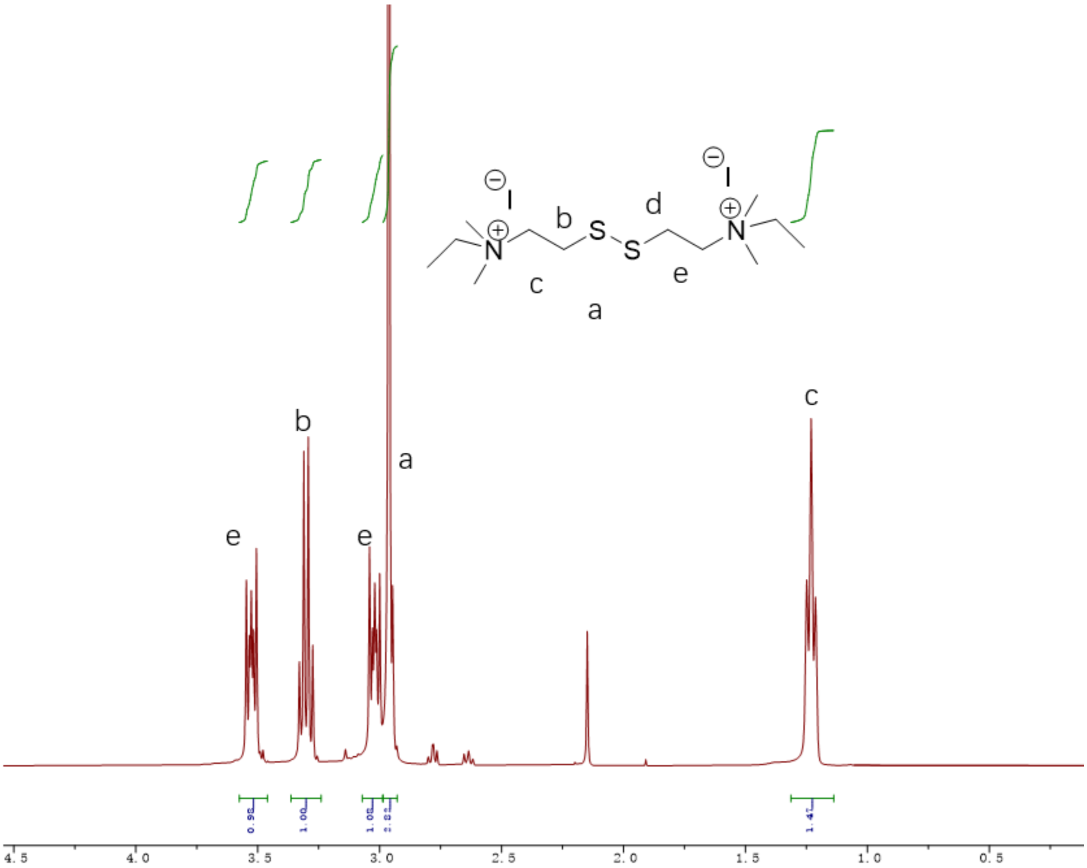

Figure S4. ^{1}H NMR spectrum of QAS-SQA.

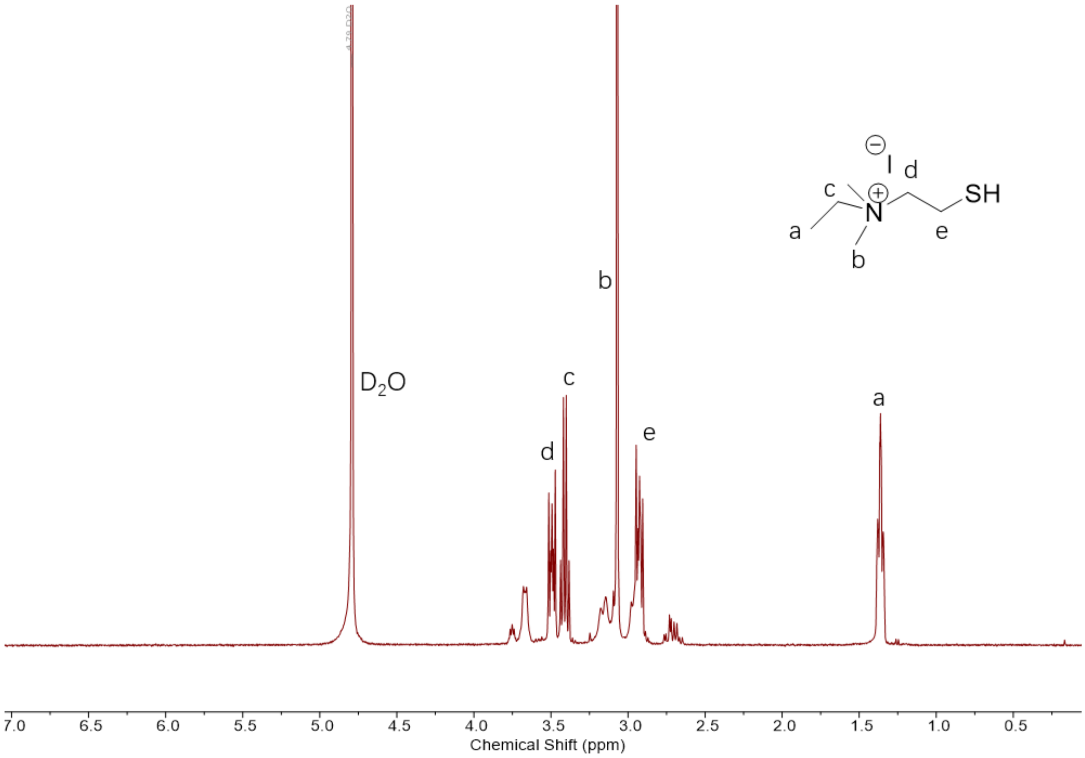

Figure S5. ^{1}H NMR spectrum of QA-SH.
